# Non-enzymatic covalent modifications: a new link between metabolism and epigenetics

**DOI:** 10.1007/s13238-020-00722-w

**Published:** 2020-04-30

**Authors:** Qingfei Zheng, Igor Maksimovic, Akhil Upad, Yael David

**Affiliations:** 1 Chemical Biology Program, Memorial Sloan Kettering Cancer Center, New York, NY 10065 USA; 2 Tri-Institutional PhD Program in Chemical Biology, New York, NY 10065 USA; 3 Department of Pharmacology, Weill Cornell Medicine, New York, NY 10065 USA; 4 Department of Physiology, Biophysics and Systems Biology, Weill Cornell Medicine, New York, NY 10065 USA

**Keywords:** epigenetics, metabolism, non-enzymatic modification, chromatin, human disease

## Abstract

Epigenetic modifications, including those on DNA and histones, have been shown to regulate cellular metabolism by controlling expression of enzymes involved in the corresponding metabolic pathways. In turn, metabolic flux influences epigenetic regulation by affecting the biosynthetic balance of enzyme cofactors or donors for certain chromatin modifications. Recently, non-enzymatic covalent modifications (NECMs) by chemically reactive metabolites have been reported to manipulate chromatin architecture and gene transcription through multiple mechanisms. Here, we summarize these recent advances in the identification and characterization of NECMs on nucleic acids, histones, and transcription factors, providing an additional mechanistic link between metabolism and epigenetics.

## Introduction

**Figure 1:**
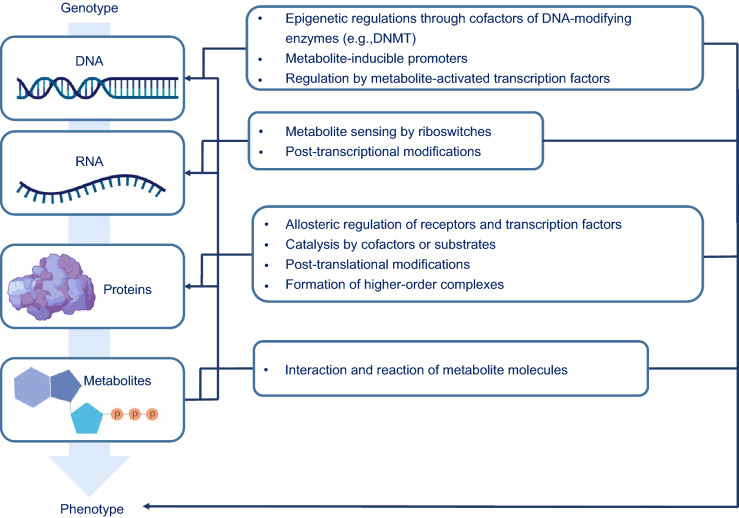
The multi-level crosstalk between metabolism and epigenetic regulation of cellular transcription

The genetic information of eukaryotes and archaea is packaged in the nucleus as a dynamic nucleoprotein chromatin complex that not only stores it efficiently but also allows it to remain readily accessible (Ammar et al., 2012). At the molecular level, the DNA strand wraps approximately 1.65 times around a histone octamer complex, which itself consists of two copies of each of the four core histones (i.e., H2A, H2B, H3, and H4) forming a nucleosome, the fundamental unit of chromatin (McGinty and Tan, 2015). Histones contain an unusually high representation of positively charged lysine and arginine residues that electrostatically interact with the negatively charged phosphodiester backbone of DNA and stabilize the nucleosome core particle (Erler et al., 2014). To regulate the interactions between histones and nucleosomal DNA or transcription factors (TFs), the histone residue side-chains are modified, typically through enzyme-mediated incorporation of metabolite molecules or cofactors, such as acetylation and methylation, and even full proteins, such as ubiquitination and sumoylation (Bannister and Kouzarides, 2011). The resulting plethora of modifications regulate cellular physiology by directly impacting chromatin structure and the pattern of gene expression, including essential enzymes involved in metabolic pathways (Janke et al., 2015). Thereafter, these enzymes can directly influence the epigenetic state of DNA, RNA and histones by balancing the biosynthesis of co-factors that serve as the co-substrates and donors for covalent modifications (e.g., S-adenosyl methionine for methylation and acyl-coenzyme A for acylation), thereby propagating the feedback loop (Fig. 1) (Rinschen et al., 2019).

Beyond enzyme-mediated epigenetic modifications, chemically reactive metabolites have been shown to directly modify nucleotides and histones via spontaneous non-enzymatic reactions (Zheng et al., 2019). Unlike canonical post-translational modifications (PTMs), non-enzymatic covalent modifications (NECMs) accumulate over time and are much more dependent on the cellular microenvironment (Harmel and Fiedler, 2018). Although metabolite-induced NECMs have lower selectivity than enzymatic modifications, histone proteins are particularly susceptible to NECMs due to their long half-lives within cells and disordered, nucleophilic tails (Commerford et al., 1982). Indeed, NECMs have emerged as a new family of chromatin modifications with direct effect on its structure and function. These NECMs have been identified on DNA, RNA and histones and are implicated in disease states; however, their pathophysiological mechanisms, particularly, the presence of any causative relationships, remain elusive (Zheng et al., 2019). In this review, we summarize recent advances in NECM characterization, categorize them based on chemical reactions, and discuss their corresponding functions in disease progression, subsequently providing new perspectives regarding the link between metabolism, diet, and epigenetic regulation.

## Glycation

**Figure 2:**
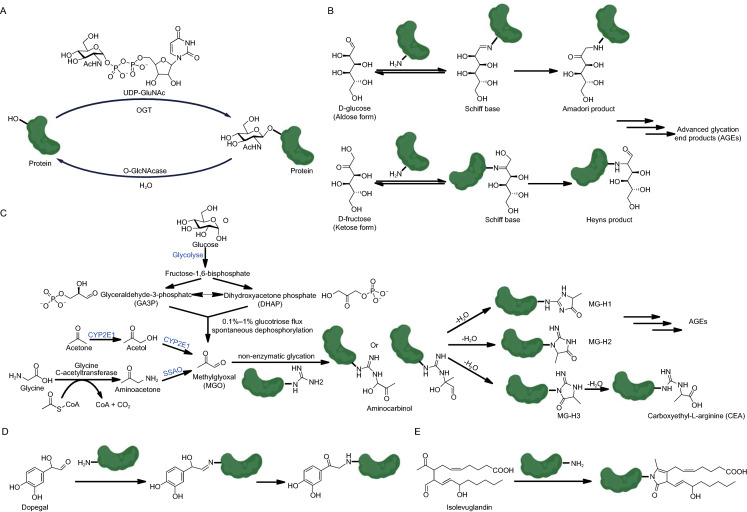
**Glycation and sugar-dependent protein PTMs**. (A) O-GlcNAc transferase (OGT)-catalyzed protein glycosylation. (B) Glucose and fructose-mediated protein glycation. (C) Generation of MGO in cells and the glycation reactions induced by it. NECMs induced by dopegal (D) and isolevuglandin (E) via the Maillard chemistry

The Maillard reaction is well known in food chemistry, where aldehyde groups of reducing sugars, most of which are aldoses (glucose, ribose, deoxyribose, fucose, glyceraldehyde etc.), react non-enzymatically with the nucleophilic groups (e.g., amine, sulfydryl and hydroxyl) of biomacromolecules such as DNA, RNA and proteins, in a process known as glycation (Hellwig and Henle, 2014). Unlike O-linked glycosylation which is regulated by O-GlcNAc transferase and O-GlcNAcase, glycation donors do not require activation by uridine diphosphate (UDP) and their modification sites on proteins are primarily lysine residues instead of serine, threonine or tyrosine (Fig. 2A) (Spiro, 2002). The glycation process is relatively slow as once the initial Schiff base is formed (Fig. 2B), a rate-limiting isomerization step is required to drive the cascade forward (Hellwig and Henle, 2014). However, upon the completion of this hydride shift, an array of rearrangement products is rapidly generated, ultimately forming chemically stable advanced glycation end products (AGEs) (Singh et al., 2001).

Besides the canonical reducing monosaccharides that can be assimilated through nutrition, several sugar metabolism intermediates are also spawned from glycation pathways within cells. For instance, methylglyoxal (MGO; C_3_H_4_O_2_) is a reactive dicarbonyl sugar metabolite that spontaneously reacts with primary amine and guanidino groups (Fig. 2C) (Schalkwijk and Stehouwer, 2020). While MGO is primarily generated as a byproduct during glycolysis (Allaman et al., 2015), it can also be biosynthesized from aminoacetone by a semicarbazide-sensitive amine oxidase (SSAO) (Obata, 2006) or oxidized from acetone by a P450 enzyme, CYP2E1 (Bondoc et al., 1999) (Fig. 2C). MGO is enriched in metabolically dysfunctional cells that overly rely on glycolysis for energy, resulting in the buildup of the glycolytic intermediates glyceraldehyde-3-phosphate (GA3P) and dihydroxyacetone phosphate (DHAP), which ultimately fragment into MGO (Allaman et al., 2015). Other types of aldehyde-bearing metabolites (e.g., ascorbic acid, dopegal, formaldehyde, 5-formylcytosine, and isolevuglandins) also form NECMs on proteins via the same Maillard chemistry (Fig. 2D and 2E) (Linetsky et al., 2007; Szende and Tyihák, 2010; May-Zhang et al., 2018; Raiber et al., 2018; Wanner et al., 2020).

### DNA and RNA glycation

**Figure 3:**
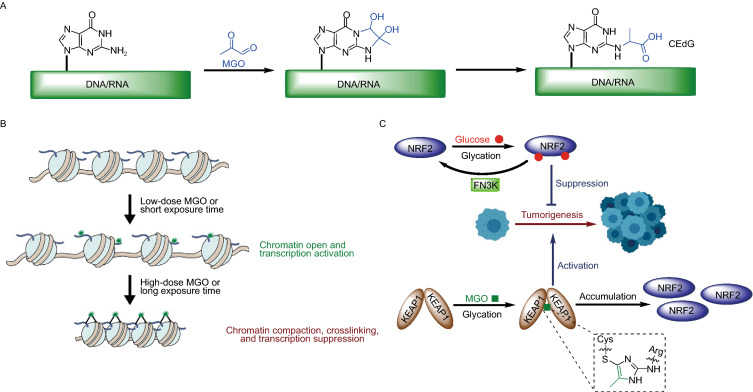
Glycation of DNA/RNA (A), histones (B), transcription factors (C), and its corresponding consequences in epigenetic regulation

Previous studies have demonstrated that guanine residues in DNA and RNA can undergo methylglyoxal glycation (Fig. 3A), thereby inducing DNA and RNA damage (Jaramillo et al., 2017; Shuck et al., 2018). The MGO-induced DNA damage product, N^2^-carboxyethyl-2’-deoxyguanosine (CEdG) (Fig. 3A), is a significant DNA AGE in human cells (~1 in 107 dG) (Synold et al., 2008). CEdG has been reported to be mutagenic in human cells and contributes to genomic instability, while this DNA damage has few corresponding repair pathways (Wuenschell et al., 2010; Tamae et al., 2011). Based on its reactivity against RNA (Mitchell et al., 2018), MGO derivatives have been applied as RNA structural probes of guanine base-pairing for transcriptome-wide RNA structure mapping (Weng et al., 2020). Finally, MGO-induced DNA/RNA glycation might be an important biomarker in human diseases such as diabetes and cancer (Jaramillo et al., 2017), however, there remains a lack of efficient sequencing methods reported for global profiling of DNA/RNA glycation sites.

### Histone glycation

Histones are primary glycation substrates because of their long half-lives and abundant lysine (Lys) and arginine (Arg) residues (Zheng et al., 2019). While different types of aldose-induced histone glycation have been observed through both *in vitro* and *in vivo* experiments for decades (Talasz et al., 2002), an epigenetic link and working model in disease states has only been recently reported (Zheng et al., 2019). Specifically, histone glycation was found to induce epigenetic dysregulation through three distinct mechanisms: 1) competition with essential enzymatic PTMs for sites (e.g., glycation adducts replace H3K4me3 and H3R8me2), 2) changing the charge states of histone tails and subsequently affecting the compaction state of the fiber, and 3) altering three-dimensional chromatin architecture by inducing both histone-histone and histone-DNA crosslinking (Zheng et al., 2019). The epigenetic impacts of histone glycation were shown to be dependent on sugar concentration and exposure time. These results were summarized in a two-stage histone MGO-glycation damage model, which proposed that the initial acute exposure stage introduces a low number of scattered adducts induces chromatin 'relaxation', transitions to fiber compaction following chronic exposure due to AGE and cross-link formation (Fig. 3B) (Zheng et al., 2019). The two-stage model intuitively suggests that histone glycation serves as a double-edged sword in gene transcription, where the compaction of chromatin is dynamically manipulated first by spontaneous rearrangement and then by crosslinking of glycation products. Despite their well-documented occurrence and effects, the detailed structures of histone AGEs are still poorly understood because of their highly dynamic nature, chemical complexity and low abundance. The most prominently used methods for characterization of histone glycation are mass spectrometry and antibody-based immunological assays (Galligan et al., 2018). However, new chemical tools (Zheng et al., 2020) and proteomics methods (Chen et al., 2019), capable of tracking or discerning specific adducts, are currently being developed to further understand the biochemical mechanisms of these events.

### Transcription factor glycation

The oncoprotein, nuclear factor erythroid 2-related factor 2 (NRF2), is a master regulator of the antioxidant response pathway and serves as a key pathological transcription factor in diseases such as cancer and atherosclerosis (Kawai et al., 2011). NRF2 exercises its functions in association with Kelch ECH associating protein 1 (KEAP1), in what is designated the KEAP1-NRF2 pathway (Kansanen et al., 2013). KEAP1 is a substrate adaptor protein for a CUL3-dependent E3 ubiquitin ligase complex which targets NRF2 for ubiquitination and subsequent degradation by the 26S proteasome (Zhang et al., 2004). PTMs on KEAP1, as well as oxidative and electrophilic stress, can reduce its ubiquitination activity, resulting in the cellular accumulation and activation of NRF2 (Keum, 2011; Kansanen et al., 2013). This in turn initiates the transcription of cytoprotective genes at antioxidant-response element loci.

Two recent studies demonstrated that both KEAP1 (Bollong et al., 2018) and NRF2 (Sanghvi et al., 2019) undergo glycation under physiologically relevant metabolic stress. The glycation of multiple lysine residues of NRF2 inhibits its oncogenic function, which is reversed by the deglycase activity of fructosamine-3-kinase (FN3K, Fig. 3C) (Sanghvi et al., 2019). Moreover, MGO selectively modifies KEAP1 to form a methylimidazole crosslink between proximal cysteine and arginine residues, resulting in the covalent dimerization of KEAP1 as well as the accumulation of NRF2 once more (Fig. 3C) (Bollong et al., 2018). These findings illustrate that sugar molecules can influence epigenetic events through glycation of transcription factors and/or their associated regulatory proteins.

### Regulatory mechanisms of glycation

Since excessive glycation forms crosslinks within chromatin, which blocks transcription, distinct pathways have evolved to ameliorate cellular glycation damage (Zheng et al., 2019). These regulatory mechanisms include preventing the initial glycation by scavenging the free reducing sugar molecules as well as directly deglycating the modified substrates. In mammalian cells, scavenger systems systematically remove most of dicarbonyl molecules while deglycases such as FN3K (Szwergold et al., 2001), PAD4 (Zheng et al., 2019), and DJ-1 (Lee et al., 2012; Richarme et al., 2015; Richarme et al., 2017) are tasked with detecting and reversing the remainder.

**Figure 4:**
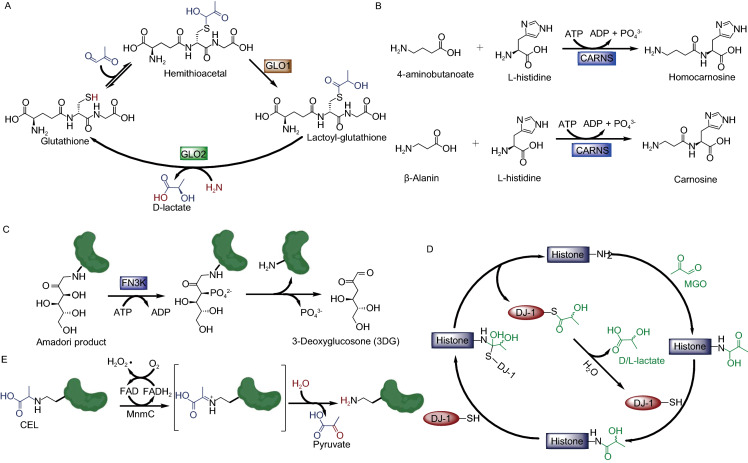
Biochemical mechanisms for antagonizing glycation by GLO1/GLO2 (A), CARNS (B), FN3K (C), DJ-1 (D), and MnmC (E)

In addition, Glyoxalases 1 (GLO1) and 2 (GLO2) together form a GLO1/GLO2 pathway that converts free MGO to D-lactate using glutathione (GSH) as a cofactor (Fig. 4A) (Xu and Chen, 2006; Distler and Palmer, 2012). First, the glutathione reacts with the dicarbonyl and forms a hemithioacetal which GLO1 can convert into lactoyl-glutathione (Distler and Palmer, 2012). GLO2 then hydrolyzes the lactoyl-glutathione, releasing D-lactate and regenerating the glutathione (Xu and Chen, 2006). Carnosine synthase 1 (CARNS1) is an ATP-dependent enzyme that catalyzes the condensation of L-histidine and β-alanine to form the dipeptide metabolite carnosine (Fig. 4B) (Drozak et al., 2010). Carnosine is an endogenous small molecule scavenger for both reactive oxygen species (ROS) and reactive carbonyl species (RCS) (Cripps et al., 2017). These scavenging mechanisms inspired the development of drug leads, such as alagebrium chloride (ALT-711), for anti-glycation and anti-aging (Little et al., 2005).

As a kinase, FN3K catalyzes the C-3 phosphorylation of fructosamines formed by glucose-glycation, and the resulting unstable phosphate product undergoes spontaneous cleavage to yield 3-deoxyglucosone (3DG) and the regenerated amine (Fig. 4C) (Van Schaftingen et al., 2007). This enzymatic activity of FN3K has been demonstrated through both *in vitro* and *in vivo* experiments, however, its mitochondrial and cytosolic subcellular localization restricts FN3K from exhibiting its deglycation function as an eraser enzyme in the nucleus (Veiga da-Cunha et al., 2006). Alternatively, peptidyl arginine deiminase 4 (PAD4/PADI4) is known to be responsible for the conversion of arginine side-chains into citrulline (Suzuki et al., 2002) and antagonizing histone arginine methylation (Cuthbert et al., 2004; Wang et al., 2004). Recently, PAD4 has been shown to be capable of reversing MGO-glycation on H3 and H4 and converting their early glycated arginine adducts into citrulline (Zheng et al., 2019). DJ-1 (also referred to as PARK7) belongs to the peptidase C56 family of proteins and protects neurons against oxidative stress and cell death (Taira et al., 2004). Its catalytic cysteine residue (C106) is positioned in a ‘nucleophilic elbow’ and responsible for both its oxidative stress sensing and deglycase activity (Nair et al., 2018). Previous studies indicate that DJ-1 erases early glyoxal (GO) and MGO-glycation adducts from both nucleotides and proteins (Fig. 4D) (Richarme and Dairou, 2017). Moreover, DJ-1 is also capable of converting free MGO into L/D-lactate through intermolecular hydrolysis of DJ-1 arginine and lysine residues that have reacted with the free MGO and formed early glycation intermediates (Toyoda et al., 2014; Zheng et al., 2019). Intriguingly, MnmC, which is involved in the bacterial tRNA-modification pathway and is FAD-dependent, was recently reported to be capable of reversing the AGEs, carboxyethyl-lysine (CEL) and carboxymethyl-lysine (CML), releasing an unmodified lysine structure (Kim et al., 2019). The engineered variant of MnmC has improved catalytic properties against CEL (Fig. 4E), thus providing insights into future protein-based therapies for AGE-induced protein damage (Kim et al., 2019).

### Glycation and human diseases

Metabolic syndromes and diabetes increase the risks associated with neurodegenerative diseases, cancer, and hypoimmunity, among other disorders (Kopelman, 2007). Aldose-induced glycation opens a new door to expound this clinical phenomenon, however, an accurate mechanistic explanation for the correlation between glycation and human disease has remained elusive. Existing efforts indicate that glycation plays important pathophysiological roles in disease progression (Fournet et al., 2018). In cancer, imbalanced glycation could promote cancer by several mechniasms; the two recent transcription factor studies uncovered completely distinct biological implications of the NRF2/KEAP1 glycation pathway in cancer development. The glucose-induced glycation of NRF2 influences its protein-protein interaction properties and suppresses its oncogenic activity (Sanghvi et al., 2019), while the MGO-induced glycation of the tumor suppressor KEAP1 causes the accumulation of NRF2 in cells and thus promotes cancer progression (Fig. 3C) (Bollong et al., 2018). The two-stage model of histone MGO-glycation (Fig. 3B) also provides a practical explanation for the observation that moderate amounts of MGO benefits cancer cell proliferation through the promotion of promiscuous transcription, while excess MGO causes chromatin crosslinking, subsequently abated transcription, and ultimately leads to cell death (Zheng et al., 2019).

In neurons, MGO-glycation of Nav1.8, a human sodium ion channel, intensifies nociceptive neuron firing and causes hyperalgesia in diabetic neuropathy (Bierhaus et al., 2012). Furthermore, for decades, AGEs have been correlated to neurodegenerative disorders such as Alzheimer’s, Parkinson’s, and Huntington’s diseases (Li et al., 2012). Interestingly, the deglycase activity of DJ-1, which is also known as Parkinson disease protein 7 (PARK7), plays an important role in the progression of a familial form of Parkinson’s disease (Repici and Giorgini, 2019). Given that core histones in neurons have extremely long half-lives due to lack in replication, one hypothesis proposes that DJ-1’s deglycase activity is a protection mechanism against the development of neurodegenerative diseases (Ariga et al., 2013).

It has been well established that diabetic patients experience significant and characteristic hypoimmunity and/or immune dysfunction (Geerlings and Hoepelman, 1999). Previous studies also showed that high plasma concentrations of aldoses or reactive carbonyls, such as methylglyoxal, are associated with obesity and diabetes (Matafome et al., 2013). One possible mechanism for the formation of diabetic hypoimmunity is that aldose-mediated glycation of immunoglobulins and surface receptors causes immunocyte exhaustion, while histone and DNA glycation may lead to long term epigenetic impacts on immune responses (Wei et al., 2017; Teodorowicz et al., 2018).

## Acylation

Acylation is a ubiquitous and important post-translational modification that regulates protein structure and function (Drazic et al., 2016). While most of the cellular protein acylations are facilitated by acyltransferases, non-enzymatic acylation induced by activated esters or anhydrides is also widely reported (Wagner and Hirschey, 2014). Importantly, some of these non-enzymatic acylation adducts were shown to be removed by common deacylases such as SIRT2 and SIRT3 (Wagner and Hirschey, 2014). Since most of the reported deacylases (such as the sirtuin family) are cofactor NAD^+^-dependent, the metabolic disorder of NAD^+^ and NADH biosynthesis will also influence the deacetylation regulations in cells (Wagner and Hirschey, 2014; Drazic et al., 2016).

**Figure 5:**
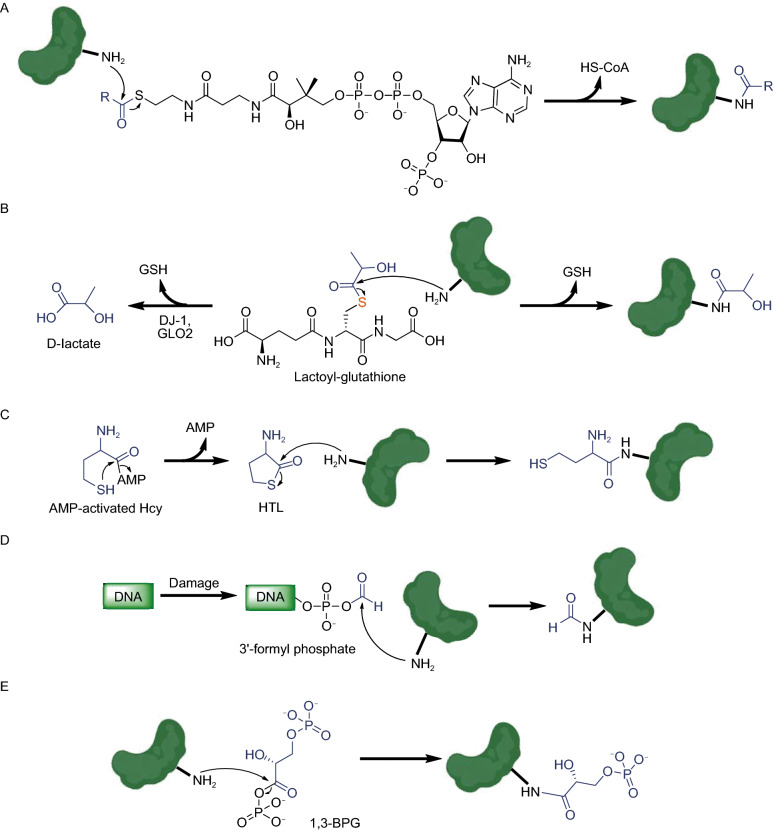
Non-enzymatic acylation induced by acyl-CoA (A), lactoyl-glutathione (B), HTL (C), 3’-formyl phosphate (D), and 1,3-BPG (E)

Coenzyme A-activated thioesters of different acids not only serve as donors for enzymatic acylation but also non-enzymatically modify proteins, generating acylated lysine residues (Fig. 5A). Recently, the GSH-activated thioester of lactate has been reported to serve as the donor for lysine lactoylation of glycolytic enzymes (Fig. 5B) (Gaffney et al., 2019) while CoA-activated lactate serves a similar function on histones (Zhang et al., 2019). The lactoylation donor, lactoyl-glutathione, can be specifically hydrolyzed by GLO2 and DJ-1 (Fig. 5B) (Xu and Chen, 2006; Matsuda et al., 2017). Interestingly, since GSH-activated lactate is biosynthesized by GLO1 from MGO (Fig. 4A) (Distler and Palmer, 2012), lactoylation is a new type of NECM indirectly induced by MGO. Homocysteine thiolactone (HTL) is an intramolecular thioester of homocysteine (Hcy), which induces non-enzymatic homocysteinylation on lysine residues (Fig. 5C) (Jakubowski, 2000). Recent studies in neuronal tissues have shown that multiple residues of all four core histones can be modified by HTL, subsequently down-regulating the expression of selected neuronal-tube closure-related genes (Xu et al., 2015; Zhang et al., 2018). This discovery provides a potential mechanistic explanation for the correlation between high maternal Hcy levels and developmental neuronal tube defects.

Anhydrides are more active than esters, making them even better donors for non-enzymatic acylation. An anhydride generated during DNA damage, 3’-formyl phosphate, serves as an acyl donor for lysine formylation (Fig. 5D) (Jiang et al., 2007). An additional anhydride, 1,3-bisphosphoglycerate (1,3-BPG), is a primary glycolytic intermediate that selectively reacts with lysine residues forming 3-phosphoglyceryl-lysine (pgK, Fig. 5E) (Moellering and Cravatt, 2013). Interestingly, pgK modifications have been demonstrated to inhibit glycolytic enzymes and accumulate on proteins generating a potential feedback mechanism for glycolysis regulation (Moellering and Cravatt, 2013).

## Alkylation

**Figure 6:**
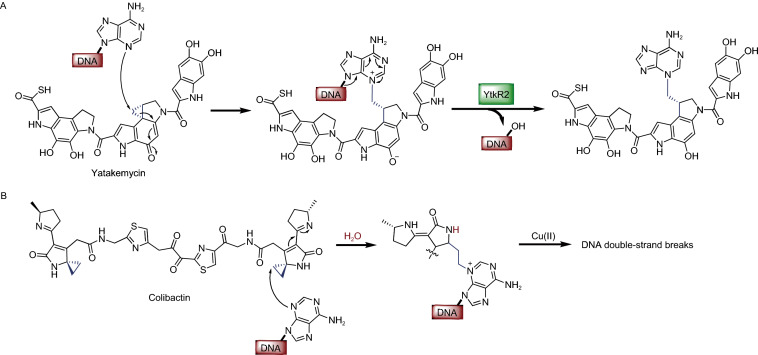
Non-enzymatic alkylation induced by microbial metabolites, yatakemycin (A) and colibactin (B)

Alkylation of proteins or nucleosides is usually induced by alkylating agents ingested from the environment, such as methylnitrosourea (MNU) and tobacco-specific nitrosamines (Shuker et al., 1993). However, the non-enzymatic alkylations induced by endogenous metabolites (e.g., S-adenosyl-L-methionine) have been reported to be potentially mutagenic reactions (Rydberg and Lindahl, 1982). Compounds with ring strain, such as the microbial metabolite, yatakemycin, which contains a unique chiral cyclopropane moiety, also exhibit alkylating activities (Parrish et al., 2003). Yatakemycin is a DNA-alkylating agent with remarkable cytotoxicity against cancer cells, and its resulting alkylation adducts can be removed by the DNA glycosylase, YtkR2 (Fig. 6A) (Xu et al., 2012). Another alkylating agent, colibactin (Fig. 6B), is a genotoxic secondary metabolite produced by microorganisms harboring the *pks* genomic island, including certain gut commensal *Escherichia coli* strains (*pks*^*+*^*E*. *coli*). Alkylation by colibactin causes multiple epigenetic impacts on the host organisms, including cell cycle arrest, DNA double-strand breaks, and senescence (Wilson et al., 2019). Moreover, colibactin-producing *E*. *coli* have been shown to accelerate colorectal cancer tumor progression, a finding that defined a new link between gut microbiota and human disease (Dalmasso et al., 2014).

## Michael addition

**Figure 7:**
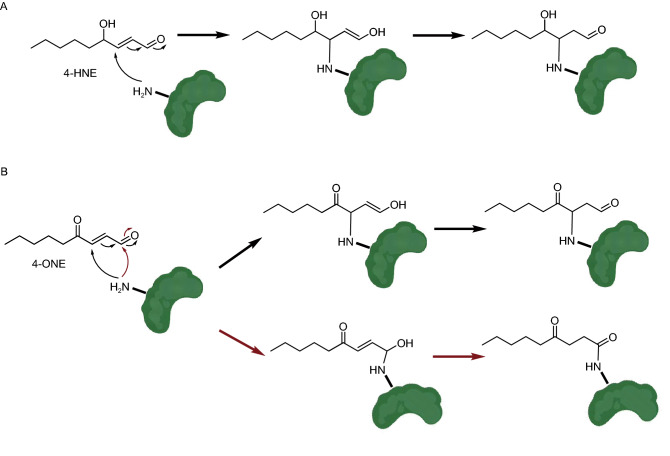
NECMs induced by 4-HNE (A) and 4-ONE (B) via the Michael addition

Enones, also termed α, β-unsaturated carbonyls, are reactive electrophilic agents that can non-enzymatically modify nucleophilic groups of proteins and nucleosides (e.g., -SH, -NH_2_ and -OH) via the Michael addition. The oxidation of cellular lipids generate α, β-unsaturated alkenals, including 4-hydroxy-2-nonenal (4-HNE) and 4-oxo-2-nonenal (4-ONE) (Doorn and Petersen, 2002; Näsström et al., 2011). These electrophilic metabolites form adducts on both DNA and histone proteins, altering the structures and functions of chromatin (Fig. 7) (Sun et al., 2017). The 4-ONE non-enzymatic addition to histones H3 and H4 was reported to prevent nucleosome assembly and occupy histone PTM sites that are essential for epigenetic regulations (e.g., H3K23 and H3K27) (Galligan et al., 2014). Similar to some acylations, the 4-ONE histone NECMs can be hydrolyzed by the deacylase, SIRT2 (Cui et al., 2017). These results suggest a connection between metabolic disorders, oxidative stress, and epigenetic regulation.

## Redox reaction

**Figure 8:**
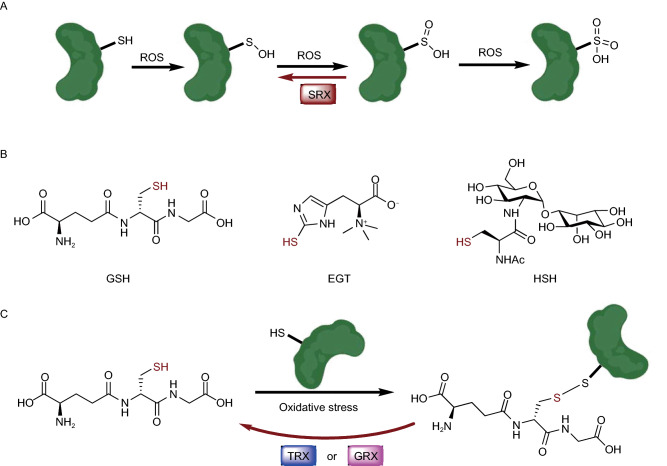
**Non-enzymatic redox reactions**. (A) Different oxidation states of cysteine side-chain. (B) Representative thiol-containing redox metabolites. (C) Glutathione oxidation of protein cysteine side-chain

Redox reactions are some of the most important and ubiquitous chemical processes in all organisms (Ochs, 2019). Reactive oxygen species (ROS), such as H_2_O_2_, are continuously produced and scavenged in cells, and can oxidize cysteine thiols into sulfenic, sulfinic, or sulfonic acid (Fig. 8A) (Chauvin and Pratt, 2017). These oxidations may often induce alterations in the structure and functions of proteins, which often act as sensors to induce a downstream cellular response to the oxidative state changes (Marinho et al., 2014). Although the precise pathological roles of ROS remain controversial (Schumacker, 2006), in human cells there are multiple oxidative stress sensor proteins including GAPDH and DJ-1, which have key regulatory cysteines sensitive to ROS fluctuations (Duan et al., 2008). Cells have evolved multiple mechanisms to complete the redox cycles, such as sulfiredoxin (SRX), a reported sulfinic acid reductase that can reduce cysteine sulfinylation (Fig. 8A) (Basu and Koonin, 2005). Moreover, cells produce metabolites possessing thiol groups to reduce ROS and protect cellular components from oxidative damage (Poole, 2015). This class of reducing agents includes among others, GSH, ergothioneine (EGT) and mycothiol (MSH), which all play critical roles in the distinct domains of life (Fig. 8B) (Hand and Honek, 2005; Van Laer et al., 2013). Intriguingly, some of these small-molecule thiols are reported to non-enzymatically modify protein cysteine residues via reduction, and this modification can be enzymatically reversed by deglutathionylase enzymes such as glutaredoxin (GRX) and thioredoxin (TRX) (Fig. 8C) (Greetham et al., 2010). For example, H3 cysteines were shown to be modified by GSH through S-glutathionylation, which leads to a looser chromatin structure (García-Giménez et al., 2013). Importantly, the levels of S-glutathionylation increase during cellular proliferation and decrease during aging, highlighting a potential physiological causal relationship between non-enzymatic redox reactions and human health (Hake and Allis, 2006).

## Conclusions and perspectives

While NECMs are long-established in biochemistry, emergent questions surrounding aberrant metabolism-related human diseases have revitalized renewed interest in them. Although membrane proteins are the primary targets of serum metabolites (Matsuda et al., 2013), core histones are the principal targets of intracellular metabolites during NECM formation (Zheng et al., 2019). Because of their long half-lives and nucleophilic N-terminal tails, histones accumulate stable enzymatic and non-enzymatic PTMs. DNA and histone NECMs, spontaneously induced by multiple classes of reactive metabolites including ROS and RCS, providing a direct causal link between metabolism and long-term epigenetic dysregulation. We propose that histones adopt the roles of ‘NECM sponges’ in cells as part of an epigenetic feedback loop in metabolic disorders.

Incidentally, some of the NECM-inducing reactive metabolites are exogenous to human cells. Metabolites produced by human gastrointestinal microbiota are known to exhibit essential functions in quorum sensing and virulence (Li et al., 2018). However, a substantial body of evidence has shown that the reactive microbial metabolites, such as colibactin (Dalmasso et al., 2014) and peptide aldehydes (Guo et al., 2017), directly modify host DNA or proteins to influence the cell cycle and immune response. The studies of reactive metabolite-induced NECMs will continue to aid in understanding pathophysiological host-microbe interactions such as the gut-brain axis (Cryan et al., 2019).

Relative to canonical and enzymatically regulated biomolecule modifications, NECMs are less characterized due to the structural diversity, dynamic nature, and instability of the adducts formed (Zhu et al., 2018). There is a critical need for novel approaches to study NECMs including the development of high-resolution trace mass spectrometry, chemical probes for specific enrichment, and site-specific antibodies. Individual and customized NECMs can also be specifically introduced into designated targets *in vivo* using intein-mediated protein splicing (Maksimovic et al., 2019) and amber codon suppression (Zhang et al., 2003).

Overall, non-enzymatic covalent modifications, which are identified as a ubiquitous biomarker on biomacromolecules, have extended the so-called ‘histone code’ (Jenuwein and Allis, 2001) and become a new link between metabolic disorders and epigenetic dysregulation. However, because epigenetic changes are heritable (Trerotola et al., 2015), cellular microenvironment-driven DNA and histone NECMs have potential implications in far-reaching processes such as embryonic development, ultimately resulting in postnatal impacts on organisms (Jawahar et al., 2015). Even though the interplay between metabolism and epigenetics has been well established in the past few years (Etchegaray and Mostoslavsky, 2016; Reid et al., 2017; Tzika et al., 2018; Montellier and Gaucher, 2019), recent studies of metabolite-induced epigenetic modifications opened a new door for understanding the missing links between them. Moreover, NECMs that target non-epigenetic proteins may also induce long-term biological effects and require further studies.
